# Electroactive Scaffolds to Improve Neural Stem Cell Therapy for Spinal Cord Injury

**DOI:** 10.3389/fmedt.2022.693438

**Published:** 2022-02-22

**Authors:** Anthea R. Mutepfa, John G. Hardy, Christopher F. Adams

**Affiliations:** ^1^Neural Tissue Engineering Keele, School of Life Sciences, Keele University, Keele, United Kingdom; ^2^Department of Chemistry, Lancaster University, Lancaster, United Kingdom; ^3^Materials Science Institute, Lancaster University, Lancaster, United Kingdom

**Keywords:** spinal cord injury, cell therapy, tissue engineering & regenerative medicine, electroactive, electrical stimulation

## Abstract

Spinal cord injury (SCI) is a serious condition caused by damage to the spinal cord through trauma or disease, often with permanent debilitating effects. Globally, the prevalence of SCI is estimated between 40 to 80 cases per million people per year. Patients with SCI can experience devastating health and socioeconomic consequences from paralysis, which is a loss of motor, sensory and autonomic nerve function below the level of the injury that often accompanies SCI. SCI carries a high mortality and increased risk of premature death due to secondary complications. The health, social and economic consequences of SCI are significant, and therefore elucidation of the complex molecular processes that occur in SCI and development of novel effective treatments is critical. Despite advances in medicine for the SCI patient such as surgery and anaesthesiology, imaging, rehabilitation and drug discovery, there have been no definitive findings toward complete functional neurologic recovery. However, the advent of neural stem cell therapy and the engineering of functionalized biomaterials to facilitate cell transplantation and promote regeneration of damaged spinal cord tissue presents a potential avenue to advance SCI research. This review will explore this emerging field and identify new lines of research.

## Introduction

**Figure 1:**
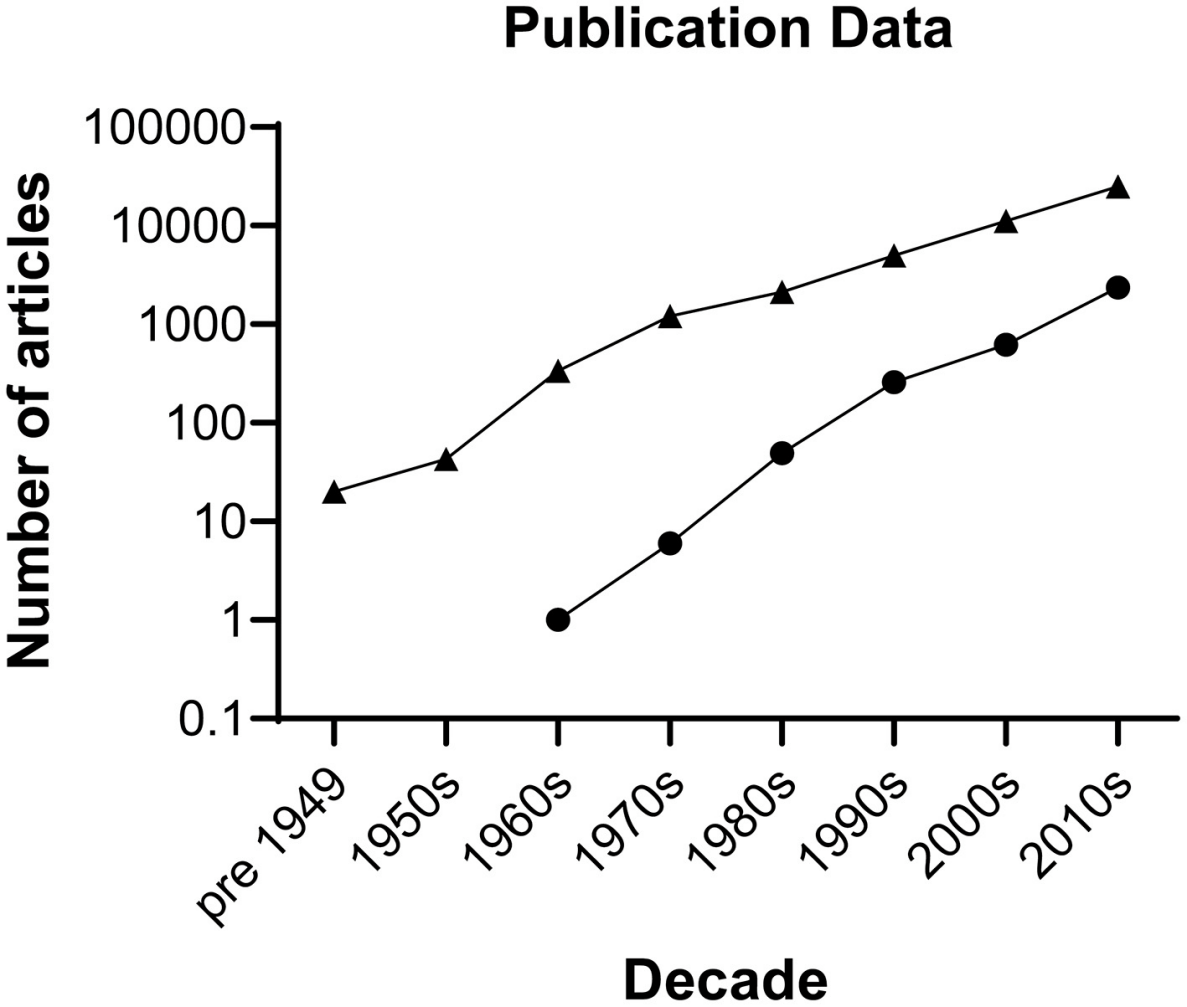
Number of publications by decade on spinal cord injury. Triangles represent hits generated searching the Web of Science repository performed on 16 December 2021 for literature describing spinal cord injury using the following keywords: (spinal cord injury) AND (spinal cord injury and imaging) OR (spinal cord injury and rehabilitation) OR (spinal cord injury and intervention) OR (spinal cord injury and surgery) OR (spinal cord injury and drug delivery) AND (spinal cord repair) AND (spinal cord injury and bioengineering) NOT (tumour) NOT (cancer). The search retrieved 42,923 articles. Circles represent hits generated searching the Cochrane Library for literature describing clinical trial articles on interventions for spinal cord injury by decade. The search retrieved 3,281 articles.

Spinal cord injury (SCI) often results in significant neurological dysfunction and long-term disability. Globally, the prevalence of SCI is estimated between 40 to 80 cases per million population per year (1), with approximately 1000 new cases per year in the United Kingdom (UK) (2). SCI is often associated with negative health and socioeconomic effects for patients, affecting young males at comparatively higher proportions than females in age-matched controls (3). Secondary complications that are widely encountered clinically include respiratory dysfunction, loss of genitourinary and gastrointestinal function, thromboembolic disease, pressure sores, neuropathic pain, spasticity and obesity (4). Furthermore, the lack of physical activity from paralysis contributes to the development of coronary artery disease, hyperlipidaemia, insulin resistance and psychosocial issues of disability in chronic SCI patients (5). The management of SCI carries substantial economic impacts with high lifetime costs averaging £1.12 million per SCI case in the UK (6). It is quite evident that the health, social and economic consequences of SCI are significant, and therefore elucidation of the complex molecular processes that occur in SCI and development of novel effective treatments is critical. To date, there has been a wealth of research into treatments for SCI albeit without successful clinical translation (Figure 1). Herein we explore the combination of stem cell therapy and electroactive biomaterials as a potential solution to this problem.

## Current Clinical Management Strategies Are Not Truly Regenerative

As no cure exists, the routine clinical management of SCI aims to prevent further injury and disability through following strategies widely recommended by clinical guidelines across multiple healthcare systems (7–9). On admission to the emergency room, evidence-based guidance recommends a clinical assessment and, often, stabilization of the spine through traction realignment surgery to prevent further injury to the spinal cord (1, 8). Radiographic evaluation and other imaging techniques such as magnetic resonance imaging, are routinely performed to map the lesion's location to define the level and severity of injury on the spinal cord and column, thereby setting surgical outcomes (10). Further surgical recommendations for SCI post realignment aim to remove fractured bone and disk fragments, foreign objects or the repair of slipped disks. Often, decompression of pressure from cerebrospinal fluid that presses on the spinal cord (decompression laminoplasty) is performed within at least 24 h post-trauma to preserve surviving neurons from blood brain barrier breach processes and reduce the risk of secondary injury (11). Several studies suggest that early surgical management in the acute phase is intrinsically connected to an earlier initiation of rehabilitation protocols and an improvement in neurological outcomes (12, 13). In the scope of recommended guidelines for SCI management, timely surgical intervention essentially serves as a neuroprotective strategy and not a predictor of full functional recovery. An alternative school of thought points to recumbence as a management option for SCI as opposed to surgical decompression. There is evidence that Active Physiologic Conservative Management (ACPM) yields neurological recovery by reducing neurological deterioration, purported to be at risk of being exacerbated by surgical management (14–16). ACPM is reported to bear advantages over costly surgery in that neuropathic pain may be minimized in patients while allowing for maximal range of motion outcomes, dependent on the injury (17). However, a Cochrane review concluded that there was insufficient evidence to determine whether surgical management bears advantages over conservative management of spinal burst fractures (18). Clearly these management options would likely require well designed clinical trials to determine the equality or superiority of each management option in relation to neurological outcomes in SCI. Crucially though, no current therapeutic route is truly regenerative and cannot restore circuitries that have been lost after SCI.

## The Complexity of SCI is a Major Challenge to Successful Repair

As outlined above there is a market need to develop new therapies which can restore neural pathways after SCI. However, this constitutes a major challenge given the complexity of the tissue and the processes that occur with associated injury. The spinal cord is part of the central nervous system (CNS) and is the major conduit and reflex center between the peripheral nervous system and the brain. Anatomically, a transverse section of the spinal cord can be divided into two sections: the white and gray matter. The white matter surrounds the gray matter and contains axons that form nerve tracts ascending to and descending from the brain. The gray matter in the center contains nerve cell bodies of both projection neurons and interneurons which form a complex circuitry to regulate neural information at the level of the spinal cord. Crucial to the function of the nervous system are the surrounding glial cells which consist of the astrocytes, oligodendrocytes, ependymal cells and microglia derived from blood. In the white matter, axons are ensheathed by myelin, produced by the oligodendrocytes. Astrocytes have many hypothesized functions including clearance and recycling of neurotransmitters at synapses; maintaining homeostasis; providing metabolic support to neural cells and responding and regulating the nervous system response to injury. Microglia are sometimes considered the immune cells of the brain and also mediate response to injury, whilst phagocytosing debris and releasing cues to surrounding cell types. Finally, the spinal cord parenchyma is separated from the blood-stream *via* a mixture of astrocytes, endothelial cells and pericytes which form the blood brain barrier (BBB). The first challenge to repairing the spinal cord then is to restore this complex cytoarchitecture essential for function.

**Figure 2:**
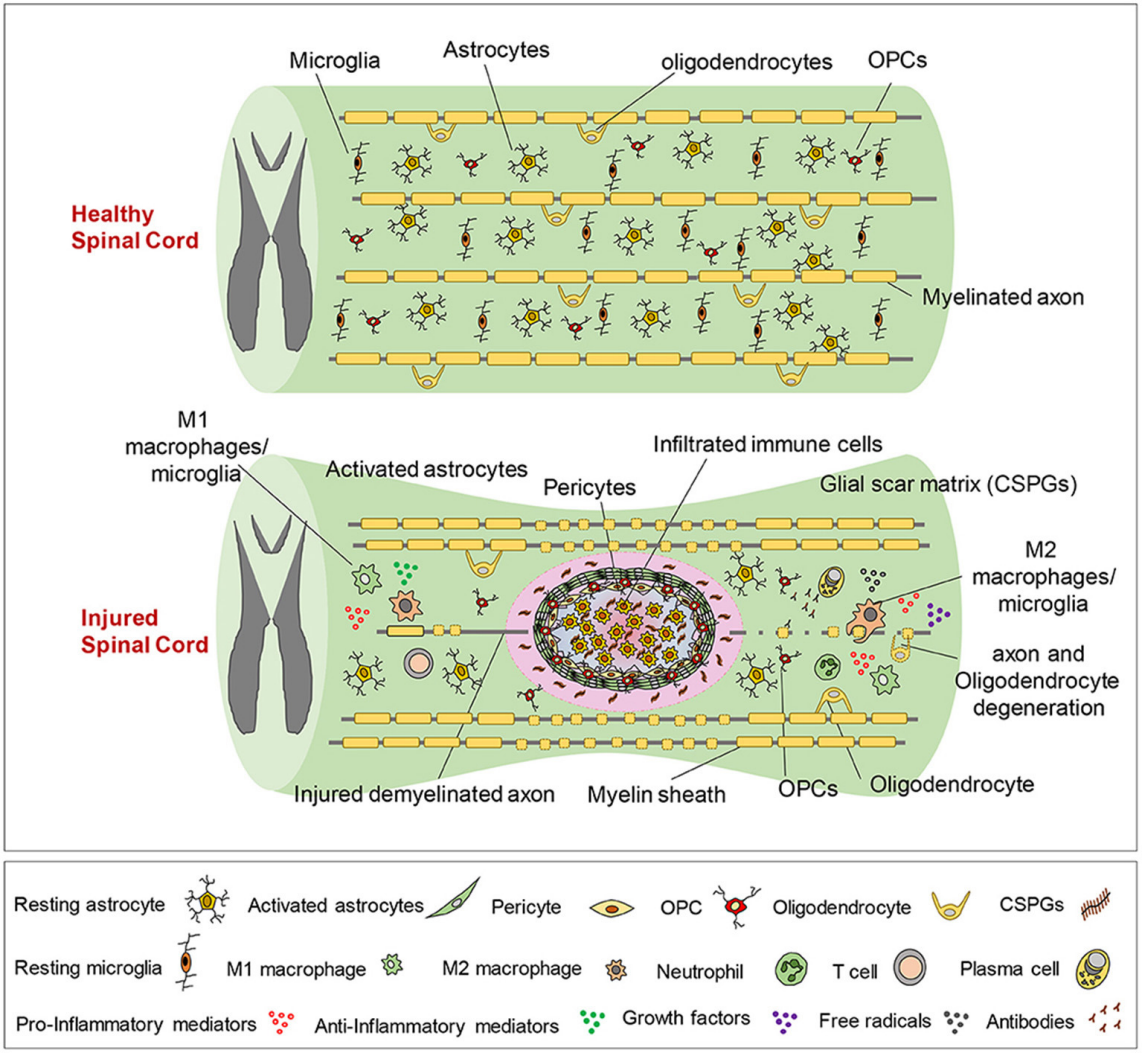
The pathophysiological processes of SCI. The healthy spinal cord is composed of quiescent astrocytes and microglia. Upon injury a series of complex events occur over time, including the infiltration of lymphocytes that initiate inflammatory processes at the lesion site. The release of proteoglycans by activated astrocytes and microglia results in the formation of a chemical and physical barrier with reactive astrocytes around the lesion core. Dystrophic growth cones form and limited axonal sprouting and remyelination occurs through to the chronic phase of injury. Reproduced from Alizadeh et al. (23) with permission from Frontiers.

The pathophysiology of SCI is also complex, characterized by the loss or degradation of motor, sensory and autonomic functions. A series of biological events have been identified that begin soon after the initial trauma (described as the primary injury process) and last for several hours, days and even up to years (contributing to what is described as the secondary injury process). From the onset of the initial trauma, blood-brain-barrier breach and tissue damage, an interdependent series of cellular and systemic events occur in the nervous, vascular and immune systems as they respond to injury, the exact details of which is still a major line of active research (19, 20). The timeline of events from the onset of injury is illustrated in Table 1. It is understood that an inflammatory cascade occurs as reactive astrocytes, macrophages, activated microglia and lymphocytes infiltrate the injury. These immune cells have been postulated to have the main function of clearing necrotic nerve fibers and myelin debris within the extracellular space of the spinal cord lesion (21). However these immune cells release pro-inflammatory cytokines, chemokines, free radicals and nitric oxide that further exacerbate secondary neuronal and glial death and subsequent necrosis during the inflammatory cascade (22). Whilst inflammation undoubtedly has an important role in stabilizing injury, there may be therapeutic opportunities for the modulation of inflammatory procedures to favor the environment for regeneration. Of the cumulative biochemical changes that occur in the secondary injury processes, the glial scar, a dynamic structure, forms over the time course from injury and largely plays a role in the inhibition of regeneration. It is evident that the glial scar contains multiple cellular components and a complex extracellular matrix (ECM) that induces a response to SCI which paradoxically inhibits regeneration whilst remodeling the spinal cord tissue to contain the injury (Figure 2). Activated astrocytes and microglia have been shown to secrete several types of proteoglycans such as chondroitin sulfate proteoglycans (CSPG), NG2 proteoglycan and phosphacan that are associated with scar tissue (24). Proteoglycans are ECM molecules which play roles in axonal plasticity, regeneration and remyelination. However, these ECM molecules form a chemical and physical barrier with reactive astrocytes around the lesion core, thereby impeding the formation of neural circuitry across the lesion (23, 25, 26). Furthermore, the distal endings of severed axons form dystrophic growth cones as they are exposed to myelin-associated inhibitors (MAI) released by necrotic oligodendrocytes, thus contributing to the scar and cavity formation (27). Some axonal sprouting, synapse formation and remyelination has been shown to occur in spared tissue through to the chronic phase of SCI, mediated by neural plasticity (28). However, the glial scar persists, hindering any endogenous regeneration of neural tissue at the injury site (29, 30).

**Table 1:** A timeline of the sequence of pathophysiological processes that occur from the onset of primary injury to secondary injury.

**Phase**	**Physiological processes**
Acute (Up to 72 hours)	- Cord oedema, intracellular swelling - Hemorrhage - Regional cord perfusion shifts - Inflammatory response: free radical production, lipid peroxidation and cytokine release - Membrane instability: shifts in electrolytes and accumulation of neurotransmitters - Demyelination - Cell necrosis and apoptosis
Sub-acute (days to weeks)	- Proximal and distal extension of oedema, necrosis and apoptosis - Continued inflammatory response - Vascular angiopathy - Peak levels of astrocyte and macrophage activity - Initial scar formation - Neuroplasticity - Spasticity
Chronic (months to years)	- Formation of fluid – filled cavity - Wallerian degeneration - Glial scar formation - Demyelination - Schwann cell proliferation - Syringomyelia - Tethered cord - Neurite sprouting, altered neurocircuits and chronic pain syndromes

*Reprinted from Mataliotakis and Tsirikos (19) with permission from Elsevier*.

Given the multiple inhibitory mechanisms present and limited intrinsic regeneration, an efficacious therapy for the successful repair of SCI would need to address all, if not most of the barriers to endogenous regeneration. The majority of experimental therapies aim to address one or two mechanisms of secondary injury processes with little success. It is therefore imperative to follow a combinatorial approach over the time course of SCI to facilitate neural tissue repair of the injured spinal cord.

## Stem Cell Therapy as the Basis for Combinatorial Therapy

As previously discussed, SCI is complex, and this is reflected to date where almost all therapies that have shown promise at the preclinical stage have failed to translate into clinically effective treatments. Clearly, a combination of therapies would ideally be required to overcome the inhibitory environment in SCI through modulating the immune response, replacing lost cells and promoting nerve fiber growth to restore neuronal connectivity. In light of the above, stem cells are of particular interest as an avenue to treat SCI. Stem cells are defined as immature cells that have the capacity to self-renew and to develop into specialized cells, that is, they can become any cell type present within an organism. A wealth of research has gone into the identification and characterization of stem cells, leading to a profound interest in their potential for the treatment of SCI, traumatic brain injury and neurodegenerative diseases (31, 32). Preclinical studies indicate that the transplantation of stem cells may contribute to spinal cord repair by: replacing lost nerve cells; generating new nerve and glial cells that can act as a bridge across the injury site to stimulate the regeneration of damaged axons; protecting the cells at the injury site from further cell death by releasing protective growth factors (33) and preventing the spread of the injury by modulating the inflammatory response mediated after SCI (34). This section will outline some stem cell candidates for SCI therapy and argue neural stem cells (NSCs) may be particularly attractive as a transplant population.

### Stem Cell Candidates for SCI Cell Therapy

A small number of ependymal cells, the endogenous stem cells of the CNS, have been characterized in the spinal canal. On review, it is believed that these endogenous multipotent cells are unlikely to contribute to any regeneration after SCI (35). The general consensus suggests that an upregulation of neurotrophic factors such as ciliary neurotrophic factor (CNTF) after injury largely promotes astrocytic differentiation of these multipotent cells. Johe et al. (36) report up to 98% of these cells differentiate into astrocytes *in vitro* through glial fibrillary acid protein (GFAP) expression analysis. Further reports have since corroborated this finding (37), which was initially discovered through pioneering research by Hughes et al. (38). In contrast, factors that promote neurogenesis and oligodendrogenesis such as brain derived neurotrophic factor (BDNF) which is largely secreted by neurons, are observed at low levels due to neuronal cell death (39, 40). The main idea from this research indicates that injury promotes the significant differentiation of ependymal cells to astrocytic cell fates which appear not to contribute to regeneration in the injured spinal cord, and, conversely contribute to the development of allodynia in SCI. The regeneration of neurons and oligodendrocytes, the other two major cell types within the CNS, is certainly crucial to reconstitute damaged tissue. Therefore, exogenous cell therapy bears the potential to replace lost neuronal and glial cells and also provide neurotrophic factors which are crucial to neural tissue repair. Recent advances in stem cell technology have translated into unlimited sources of neural progenitors and glial cells for cell based therapy. Olfactory ensheathing cells (OECs), NSCs, mesenchymal stem cells (MSCs), embryonic stem cells (ESCs) and induced pluripotent stem cells (iPSCs) have been widely assessed as cell sources for SCI cell therapy. OECs, albeit not stem cells, have been studied in neural tissue repair applications and shown to promote the growth of olfactory receptor neurons, and regenerate axons in pre-clinical SCI models (41). Nakhjavan-Shahrak et al. (42) conducted a meta-analysis to determine the efficacy of OEC transplantation across several preclinical studies and clinical trials, yielding some limitations. It appears that a significant limitation of this cell therapy appears to be the lack of effect on allodynia and possibility of aggravating hyperalgesia, despite significant motor function improvements reported consistently across preclinical studies (43–47). Whilst trial data largely concludes the feasibility and clinical safety of OEC therapy in SCI patients, reproducible and robust functional recovery has not been reported (48–50). Alternatively, MSCs indicate moderate therapeutic efficacy for the treatment of various neurological diseases and can be acquired from multiple autologous and allogeneic sources (51). MSCs can self-renew, differentiate into several lineages and have been shown to participate in immunomodulation that may reduce secondary injury and enhance remyelination and axonal regeneration in SCI (52–55). However, it is evident that further developments are required to determine the optimal source of MSCs for SCI and to determine the extent in which MSCs can replace neurons. “Neuron-like” cells have been generated from MSCs and characterized with variable purity and heterogeneity post-differentiation across several reports in the literature (55). Interestingly, a comparative study of bone-marrow derived MSCs (BM-MSCs), neural progenitors derived from a spinal fetal cell line (SPC-01) and iPSCs suggested the latter as the efficacious cell candidate for SCI cell therapy in rat SCI models (56). Such findings correlate with previously reported limitations of MSCs, attributed to improved graft survival, reduction of glial scarring, tissue sparring and increased axonal sprouting in iPSCs as reported by the authors. The current clinical trial data consistently indicates the safety of MSCs, however trials have failed to progress to Phase III due to findings of modest clinical efficacy, despite promising clinical developments underway (57–61). Indeed, there are reports of a combinatorial approach where the co-transplantation of multiple cell candidates, including MSCs, OECs and NSCs, has been tested in a bid to develop an efficacious cell therapy with synergistic cell properties for SCI repair (62–64). The outcomes are variable, although on review appear promising as a growing number of studies are following this approach in cell therapy applications for neurological diseases (65). However, a cell candidate that can enhance the key aspects of regeneration including cell survival, engraftment and migration into the lesion and provide neuroprotection may be the most viable strategy.

### NSCs Are a Key Transplant Population for Repairing SCI

**Figure 3:**
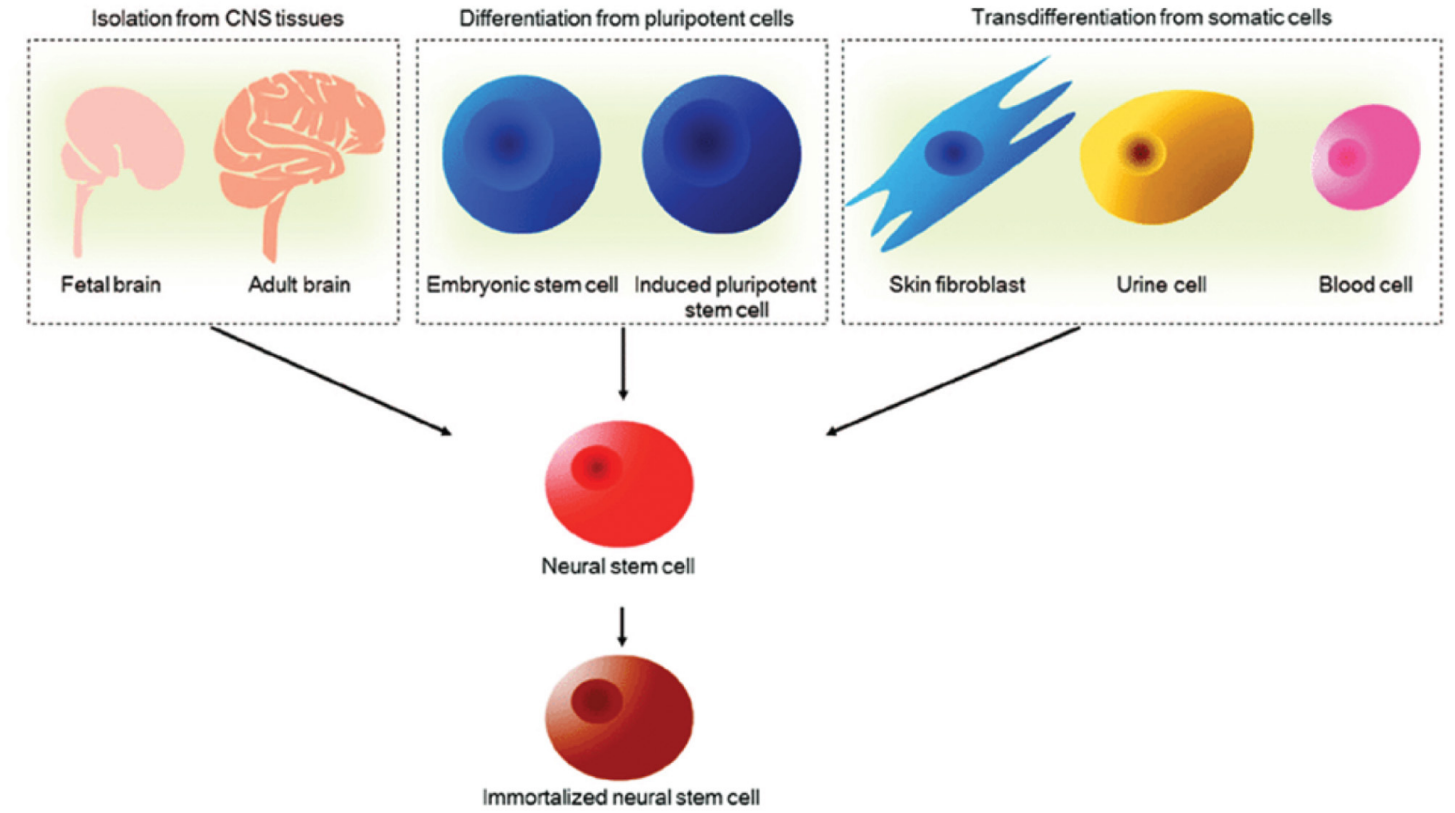
Sources of NSCs. NSCs can be derived from primary CNS tissues such as fetal and adult brain, pluripotent embryonic stem cells and induced pluripotent stem cells and transdifferentiated from human somatic cells. NSCs can be further immortalized to facilitate long-term culture for cell therapy applications. Reproduced from Tang et al. (74) with permission from Springer Nature.

NSCs are multipotent cells that can self-renew and generate all the specialized neural cells within the spinal cord, that is, neurons, astrocytes and oligodendrocytes. NSCs are capable of surviving, migrating and differentiating into the aforementioned major cell types of the CNS to promote regeneration (66–68). The trilineage differentiation potential of NSCs is a remarkable feature following demyelination and cell death induced by SCI. For example, facilitation of remyelination by transplantation of exogenous NSCs, the myelinating cells of the CNS, has been reported across many studies (27, 69, 70). Furthermore, NSCs have been shown to secrete a large number of soluble factors including neurotrophins such as BDNF, CNTF, glial cell derived neurotrophic factor (GDNF) and nerve growth factor (NGF) across several studies (71). Schubert et al. (39) have since characterized the proteins secreted by NSCs. BDNF in particular, is associated with the survival and proliferation of damaged neurons to promote an environment permissive to growth. Astrocytes have also been shown to secrete some neurotrophic factors for axonal remodeling and plasticity, as well as homeostatic support functions relevant for spinal cord repair (72, 73). Such neural precursor cells may be derived from pluripotent stem cells, primary CNS tissue and potentially from human somatic cells once a robust and ethical protocol for transdifferentiation is developed as illustrated in Figure 3 (75–80). Pluripotent stem cells carry some ethical concerns and have been shown to carry karyotypic abnormalities and a risk of tumorigenicity (81, 82). Induced Pluripotent Stem Cells (iPSCs) in particular have lower ethical implications and immunogenic concerns as they can be derived from patient-matched sources and collected using non-invasive methods. It must be noted that the potential for genetic defects, tumorigenicity and immunogenicity of transplanted cells have largely hindered the successful clinical translation of iPSC-derived cell therapies for SCI (83). An accumulation of evidence for the safety of iPSC cell therapy is actively progressing for applications in the CNS. For example, efficient techniques to differentiate and modify cells prior to transplantation are being investigated in a bid to reduce the risk of teratoma formation in preclinical models (84, 85). Such developments have continued to emerge where Khazaei et al. (86) demonstrated the possibility of enhancing neuronal differentiation and even improving motor function in rodents through counteracting Notch signaling by expressing GDNF in transplanted NPCs, thus indicating potential to enhance the safety and efficacy of iPSC derived NSC therapy for SCI. Further hurdles exist, such as lineage control in neuronal differentiation, which is out of scope of this review.

Preclinical research largely reports significant functional improvements when NSCs are transplanted into SCI models. Based on their potential advantages, NSCs have been tested in phase I/IIa clinical trials for SCI cell therapy, which have been identified in the literature and listed below:

- Shin et al. (87), *n* = 19
- Ghobrial et al. (88), *n* = 5
- Curtis et al. (89), *n* = 4
- Levi et al. (90), *n* = 31

The low number of studies and small sample sizes across the studies invariably limits the quality of evidence for safety and efficacy outcomes for NSC therapy on review. Whilst a limited number of phase I/IIa clinical trials currently exist, Tiwari et al. (91) conclude the safety of NSC transplantation for SCI in humans in a systematic review. The conclusions from the systematic review largely indicate the safety and tolerability of NSC transplantation, albeit the lack of a quantitative assessment of adverse events and serious adverse events across the studies. Furthermore, clinical efficacy reports appear to show modest improvements in functional recovery. Such promising findings are highlighted by Shin's group whom reported positive motor and sensory outcomes from their phase I/IIa clinical trial when fetal cerebral NSCs were transplanted into 19 traumatic cervical SCI patients (87). There was no evidence of tumor formation, exacerbation of neurological deterioration or neuropathic pain and spasticity, thus warranting further investigation of NSCs as SCI cell therapy candidates. Another promising insight indicates that neural stem/progenitor (NSPC) grafts can successfully integrate into spinal cord lesions and generate extensive neuronal relays across the lesion and host axon regeneration into the lesion (92–94). Ceto et al. (95) have since expanded these findings by assessing the synaptic architecture of grafted NSCS. An important finding is the formation of functional synaptic connections to corroborate the functional improvements observed in NSC grafts through animal SCI studies.

Alongside their regenerative benefits, NSCs can also be isolated and rapidly expanded as neurospheres in culture through well-established protocols (96), to generate the numbers required for transplantation. They can also be manipulated *in vitro* to potentially improve cell transplantation, for example, through genetic engineering (97, 98). However, challenges still exist, some of which are generally consistent with other cell therapy candidates for SCI repair.

## Challenges for NSC Transplantation in SCI

While exogenous NSC transplantation offers promise in reconstituting the architecture of the damaged spinal cord and promoting regeneration as discussed in the previous section, challenges for clinical translation have been encountered. Two key challenges are (i) the low survival and lack of retention of viable cell transplant populations at the injury site and (ii) the inability to control differentiation fates post transplantation as NSCs largely differentiate into astrocytes under pathological conditions. For example, Webber et al. (99) transplanted rat fetal NPCs into the dorsal column lesion site of adult rats and examined the survival of transplanted cells at 24h, 1 week, 2 weeks and 6 weeks after injury. Only minor sensory function improvement was observed and no motor function recovery was observed. The group suggested this to be the result of a high differentiation rate (40%) of grafted stem cells into glial cells as only 8% displayed neuronal morphology post-transplantation. Other reports indicate that recruited NSCs largely differentiate into astrocytes and, to a lesser degree, oligodendrocytes, but without evidence of neurogenesis after injury (100). However, Piltti et al. (101) observed predominantly oligodendrocytic differentiation when NSCs were transplanted into host parenchyma, and higher astrocytic differentiation in lesion-site transplantation in contusion injury rats suggesting the gross influence of the injury microenvironment on cell fates *in vivo*. Earlier studies concluded that NSC transplantation is associated with increased allodynia in animal behavioral studies, purported to be linked to the high astrocytic differentiation and maladaptive plasticity (102, 103). Thus a rationale to affect some control on cell fates in cell transplant populations could be important to improve functional outcome. Further challenges observed are the poor migration of the grafted cells and directionally guided axonal growth through the inhibitory microenvironment of the lesion and scar tissue (104). In light of the challenges of cell transplantation and the outcomes of NSC therapy in recent trials, tissue engineering presents as a promising avenue to address the limitations of cell differentiation fates, survival and integration of transplant populations to effect functional recovery.

### Biomaterial Encapsulation of NSCs Could Improve Regeneration

Implantable biomaterials present as a viable solution to the limitations of cell therapy in SCI as they hold potential for combinatorial therapy. Biomaterials can function as carrier vehicles for encapsulated stem cells in order to enhance survival and engraftment at the site of transplantation as reported in injury models in the CNS. Previously, biomaterials have been functionalized as drug delivery vehicles and bioactive molecule carriers, for example, to facilitate NSC survival and promote significant aligned axonal growth through BDNF and growth factor cocktail incorporation in SCI models (105–107). Furthermore, biomaterials have been stiffness-matched to CNS tissue which appears to favor cell attachment and reduce inflammatory and immune responses, conducive to cell survival and regeneration in the inhibitory microenvironment of the injured spinal cord (108–111). There is a large body of research concerning the use of implantable materials to aid transplantation of cells into sites of neurological injury. This has been well-covered elsewhere (112–114). Significant functional recovery outcomes have been observed consistently at the preclinical stage when NSCs have been delivered *via* a supporting scaffold matrix over the last decade (115–117), and yet to be clinically translated. Meanwhile, clinical developments are underway regarding the safety and feasibility of implantable biomaterials for CNS repair. Xiao and colleagues report the feasibility and safety of transplanting NeuroRegen, an implantable collagen scaffold in complete chronic SCI patients, albeit the small sample size in the study (118). However further developments are required before progress can be made toward an efficacious implantable cell therapy for SCI. Design strategies for biomaterials have certainly advanced in the last decade: biomaterials can be tailored to enhance neural regeneration by modifying physical properties in the biofabrication process through 3D bioprinting and electrospinning technologies. Developments in electrospinning technology bear potential to enhance the anisotropic extension of neurites and neuronal differentiation when cultured as a substrate for NSCs, critical properties for CNS tissue regeneration (119). Alongside myriad advantages of biomaterial mediated cell transplantation, an emergent area of biomaterial design for neural tissue repair integrates electric conductivity and electric stimulation (ES) which have been shown to influence neurogenesis, proliferation, migration, cell-cell interactions and the modulation of synapse formation (120–124). We believe there may be specific advantages to improving the regenerative potential of NSCs by encapsulation in electroactive biomaterials for implantation, which is where we will focus the next parts of the review.

### Rationale for Incorporating Electrical Stimulation Into Cell Transplantation Strategies and Challenges

**Figure 4:**
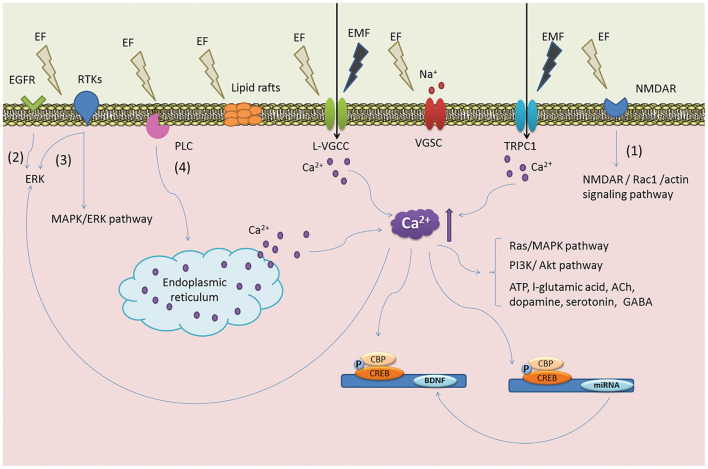
Postulated mechanism of ES on NSCs. The illustration denotes key signaling events that are thought to occur *via* ES. ES is believed to induce the reorganization of cytoskeletal filaments and lipid raft structures become polarized to initiate ERK signaling pathways and the upregulation of BDNF. These events are linked to the proliferation of NSCs and early neuronal differentiation. Reprinted from Zhu et al. (123) with permission from Elsevier.

Over the years, researchers have identified how endogenous electric fields play a critical role in the developing CNS and pathophysiological states since Luigi Galvani's landmark bioelectricity experiment (125). The presence of endogenous electric fields in the developing CNS has been well characterized. Ionic currents have been detected in the developing nervous systems of vertebrates (126). Furthermore, the disruption of the electric fields induced by ionic currents has been linked to developmental abnormalities in early pioneering studies (127). Endogenous electrical fields have been recorded during development and following injury, affecting the orientation of astrocytes and neurons, proliferation and neurite outgrowth of neurons and glia which has been demonstrated *in vitro* and in a rat sciatic nerve injury model (123, 128, 129). Similarly, early peripheral nerve studies report the benefits of delivering ES to transection injury models in adult rats. For example, ES significantly enhanced dorsal root ganglion (DRG) sensory neurons to regenerate axons after femoral trunk transection and surgical repair. This correlated with an increase in expression of growth-associated protein 43 (GAP-43) mRNA in the regenerating neurons (130). Further evidence in the literature suggests that ES upregulates neurotrophic factor release, which is essential to regeneration (Figure 4). Wenjin et al. (131) reported an increase in BDNF expression in spinal cord neurons after brief electrical stimulation for 1 h at 20 Hz after sciatic nerve transection compared to control and untreated sciatic nerve transection groups. These findings corroborated earlier work by Al-Majed et al. (132) in rat femoral neurons. In order to fully realize the potential of ES for SCI, a better understanding of the mechanisms through which ES enhances neural plasticity is warranted. Whilst peripheral nerve injury studies have largely informed the literature on SCI induced neuropathic pain, Vivó et al. (133) report enhanced axonal regeneration and functional sensory outcomes when ES was administered immediately after nerve injury in a sciatic nerve injury model. Such observations are promising and indicate the potential role that ES may play in facilitating the regeneration of nervous tissue in SCI, however there are limitations of the concept in this developing area of cell therapy, pertinent to CNS repair.

The first challenge would be in the design of a strategy that is minimally invasive and clinically safe to provide ES. The delivery of localized ES in the injured spinal cord *via* implanted electrodes has been reported preclinically with promising findings for functional recovery outcomes that have led to the growing interest in ES for SCI (130, 131, 134–137). However, there are reports that implanted stiff materials can cause local scarring from the mechanical trauma and immune reactions in the CNS (138). Previously, Potter et al. (139) demonstrated a localized inflammatory response after microelectrode implantation in the CNS using immunohistochemical markers. Furthermore, the group demonstrated an accumulation of reactive oxygen species surrounding implanted microelectrodes which were thought to impact the viability of neural tissue adjacent to the implanted electrodes (138). The build-up of scar tissue at the neural tissue interface has been shown to reduce the efficiency of the recording and stimulating capacity of implanted electrodes, introducing a challenge that has led to further progression of neural interface research and growing interest in nanomaterial science (140). Alternatively, external stimulation is a minimally invasive method of delivering ES to transplanted cells albeit with low spatial resolution and some off-target effects such as tissue scarring around electrodes in *in vivo* models (58, 141). A systematic review of the efficacy of external or transcutaneous stimulation indicates the need for testing in large clinical trials so far (142). In summary, ES has been shown to improve cell regenerative responses (of both transplanted cells and cells at the injury site) but faces challenges in efficiently detecting and supplying charge to and from neural tissue with minimal signal attenuation, immune responses and undesirable chemical reactions.

### Hybrid Electroactive Biomaterials: Combining Conductive Biomaterials With Stem Cell Therapy to Maximize Cell Transplantation Outcomes

To address the challenges outlined above, the functionalization of electroactive biomaterials presents as a promising strategy to deliver cells and modulate their behavior post-transplantation. The concept of an electroactive implantable cell transplant device has been well studied with promising reports (129, 143, 144). A vast library of electroactive biomaterials have been classified through rapidly developing fabrication technologies in regenerative medicine research, summarized in Table 2. Some well-studied electroactive biomaterials have been engineered from materials such as conductive polymers, carbon nanotubes (CNTs) and graphene for CNS repair, highlighted in this review (Table 3). Polymeric biomaterials have so far proven to provide mechanical support and potential to interact electrically with neurons in neural tissue engineering applications. Naturally occurring polymers, such as the ECM matrix protein collagen, can be fabricated with synthetic conductive polymers to form three-dimensional (3D) composite polymeric scaffolds with controlled chemical and physical properties (165). Several non-cytotoxic synthetic conductive polymers, including polypyrrole (PPY), Poly(ethylene glycol) diacrylate (PEGDA) and poly(lactic-co-glycolic acid) (PLGA), are currently being studied for the development of electroactive scaffolds for nerve injury applications (Table 3). An important aspect to inscribe is that electrical conductivity and electrical stimulation are defined as separate parameters. Coupled with ES, cells grown on electroactive scaffolds are consistently observed to possess significantly enhanced regenerative features which may improve cell therapy outcomes in SCI.

**Table 2:** A general classification of commonly studied electroactive biomaterials and fabrication methods applied in regenerative medicine applications.

**Class of electroactive material**	**Fabrication technology**	**References**
Conductive polymers PPY, PANI, PLGA, PEGDA, PEDOT	∙ Electrospinning ∙ 3D printing ∙ Freeze drying ∙ *In situ* polymerization ∙ Vapor-phase polymerization ∙ Solvent casting	Liu et al. (145) Wang et al. (146) Pelto et al. (147) Distler et al. (148) Shah et al. (149)
Carbon-based materials Carbon fibers, carbon nanotubes (CNTs), graphene and graphene oxide	∙ Electrospinning ∙ Pressure-Activated Microsyringe (PAM) ∙ High pressure carbon monoxide conversion synthesis	Chen et al. (150) Magaz et al. (151) Kim et al. (152) Li et al. (153) Guo et al. (154)
Metal- based materials Gold, Platinum, Zinc	∙ Electrospinning ∙ Capillary force lithography ∙ Electron beam evaporation	Baranes et al. (155) Wickham et al. (156) Aydemir Sezer et al. (157)

**Table 3:** A summary of some major findings in the investigation of electroactive scaffolds for neural tissue engineering.

**Study name**	**Biomaterial**	**Shape**	**Model and study system**	**Results**
Stewart et al. (121) Zhou et al. (158)	PPY Polyphenol-tannic acid- PPY	3D films 3D hydrogel	*In vitro*- hNSCs *in vitro* and *in vivo*- NSCs	Enhanced neuronal and glial differentiation following ES. Increased neurite growth and branching Enhanced neuronal differentiation on substrates with higher conductivity. Endogenous neurogenesis and significant locomotor function recovery
Lorite et al. (159) Lee et al. (160) Pan et al. (161) Qi et al. (162) Liu et al. (163) López-Dolado
et al. (164)	CNT MWCNT- PEGDA GO- PLGA GO- PLGA Silk fibroin/ graphene composite Reduced GO	3D micropillars 3D scaffold 3D electrospun nanofibres 3D electrospun nanofibres 3D scaffold 3D scaffold	*In vitro*- NSCs *in vitro*- NSCs *in vitro* and *in vivo*- NSCs *in vitro*- NSCs *in vitro*- rat spinal cord neurons *in vivo*-Spinal cord hemi-section at C6 in rats	CNTs guide neurite growth and support the long-term survival of human NSCs Enhanced NSC proliferation and early neuronal differentiation. ES enhanced neuronal maturity Enhanced NSC proliferation and neuronal differentiation *In vitro*. Effective immobilization of IGF-1 and significantly improved functional locomotor recovery, reduced cavity formation and increased neuron numbers *in vivo* Enhanced neuronal survival, differentiation and effective immobilization of IGF-1 Aligned silk fibroin/graphene scaffolds are not cytotoxic to rat spinal cord neurons and enhanced neurite outgrowth Enhanced collagen scaffold infiltration and immunomodulation. Angiogenesis and axon growth inside scaffold

*MWCNT, multi-walled carbon nanotube; PEDGA, Poly(ethylene glycol) diacrylate*.

### Molecular Mechanisms for Improved Regenerative Responses of Cells on Electroactive Surfaces

**Figure 5:**
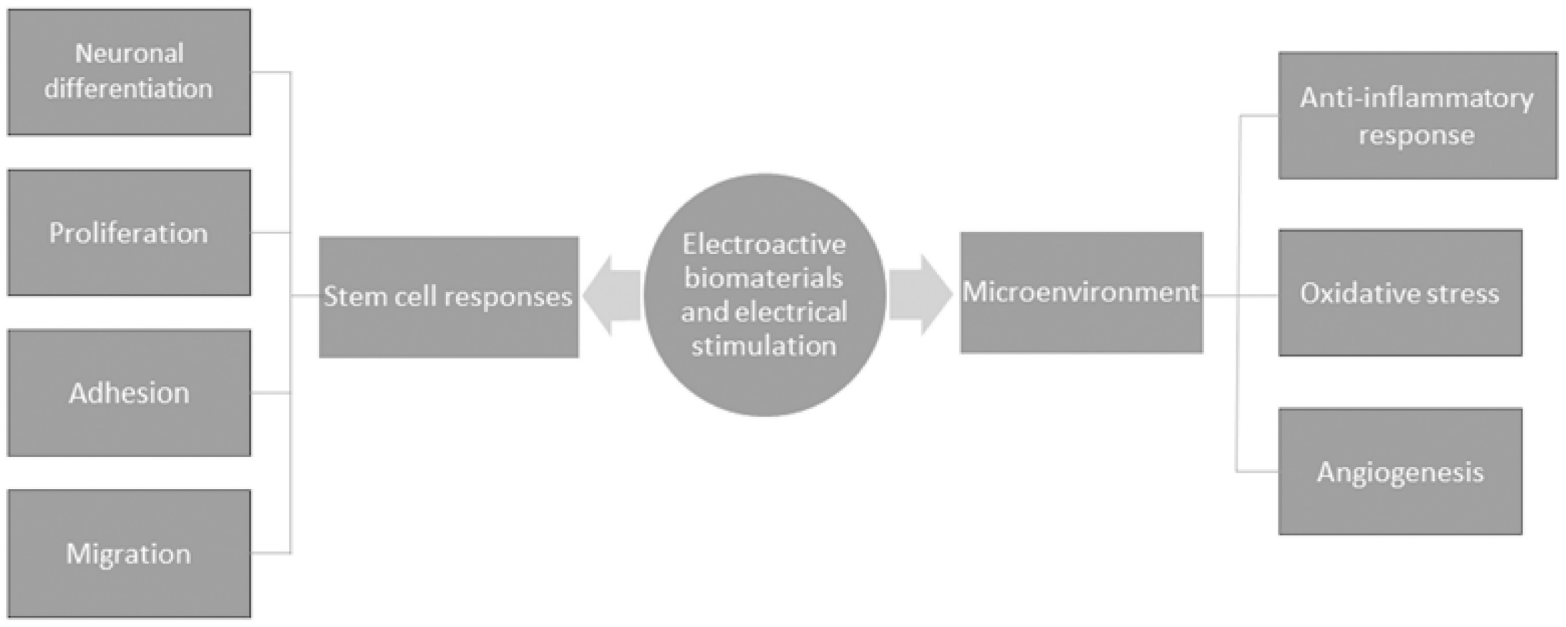
A schematic of the general mechanisms by which electroactive biomaterials and electrical stimulation enhance nerve healing.

It is well understood that neuronal cells are electroactive, and given the discovery of endogenous EFs in the developing CNS, the promotion of bioelectrical signal transmission would seem crucial for the functional restoration of damaged tissue after SCI. Electroactive scaffolds bear the advantage of enhancing the complex electrical transmission function of neuronal networks during cell-to-substrate and cell-to-cell interactions. Material scientists have made insights into how cells probe their surroundings within biomaterials by investigating conductivity as a biophysical cue, which seems to be a promising strategy to promote a well maintained homeostatic microenvironment that is tailored to enhance regeneration. In a bid to understand the mechanisms of electroactivity on nerve healing, electroactive materials are understood to regulate cellular responses to enhance regeneration. The cellular responses reported include cell adhesion, neuronal differentiation, migration and proliferation. Furthermore, electroactive materials have been shown to affect the microenvironment by modulating immune responses and oxidative stress, seemingly relevant for altering the inhibitory microenvironment of the injured CNS (Figure 5). Indeed, such anti-inflammatory responses and the regulation of oxidative stress have been reported across several studies (166–168). In consideration of the latter, the molecular mechanisms that have largely been proposed to affect nerve healing appear to do so primarily through enhanced survival, proliferation and neuronal differentiation, described in several reports in the literature.

Recently, Eftekhari et al. (169) demonstrated the capability of a novel conductive chitosan/polyaniline hydrogel in modulating neuronal differentiation through the upregulation of the MAP2 gene in rat adipose-derived stem cells at the material-tissue interface. Notably, conductivity and cell-imprinted topography were investigated simultaneously as biophysical cues to enhance the potential for neural regeneration. Many groups have corroborated the latter findings on investigating electroactive materials for NSC therapy. Stewart et al. (121) successfully induced human NSC differentiation on PPY with an electrode device. The group reported that ES of PPY induced human NSCs to predominantly β-III Tubulin (Tuj1) expressing neurons, with lower induction of glial fibrillary acidic protein (GFAP) expressing glial cells compared to controls. A morphological analysis revealed longer neurites and significant branches on stimulated cells compared to controls. Furthermore, the cultures were observed to possess clusters of neurons with longer neurites compared to unstimulated cultures. The study highlights the developments made toward directing the differentiation fates of NSCs to neuronal lineage *via* an implantable electroactive scaffold when considering a pioneer study by Schmidt et al. (170). The group reported the culture of PC-12 neuron-like cells on oxidized PPY films which were subjected to an electrical stimulus. The group reported a significant increase in neurite lengths compared with controls that were not subjected to electrical stimulation. Similarly, Moroder et al. (171) analyzed morphological changes in PC-12 cells and reported significant increases in the percentage of neurite bearing cells, neurites per cell and neurite lengths of PC-12 cells in the presence of ES compared with no ES on conductive polymer scaffolds. Notably, these studies employed cell lines, and recent preclinical developments appear to heavily report on cell lines for SCI repair applications (169, 172, 173). Whilst cell lines have their advantages in mitigating ethical implications arising from ESCs or fetal-derived NSCs, the move to physiologically relevant NSCs in CNS injury preclinical models would seem to be concomitant with clinical translation for SCI.

**Figure 6:**
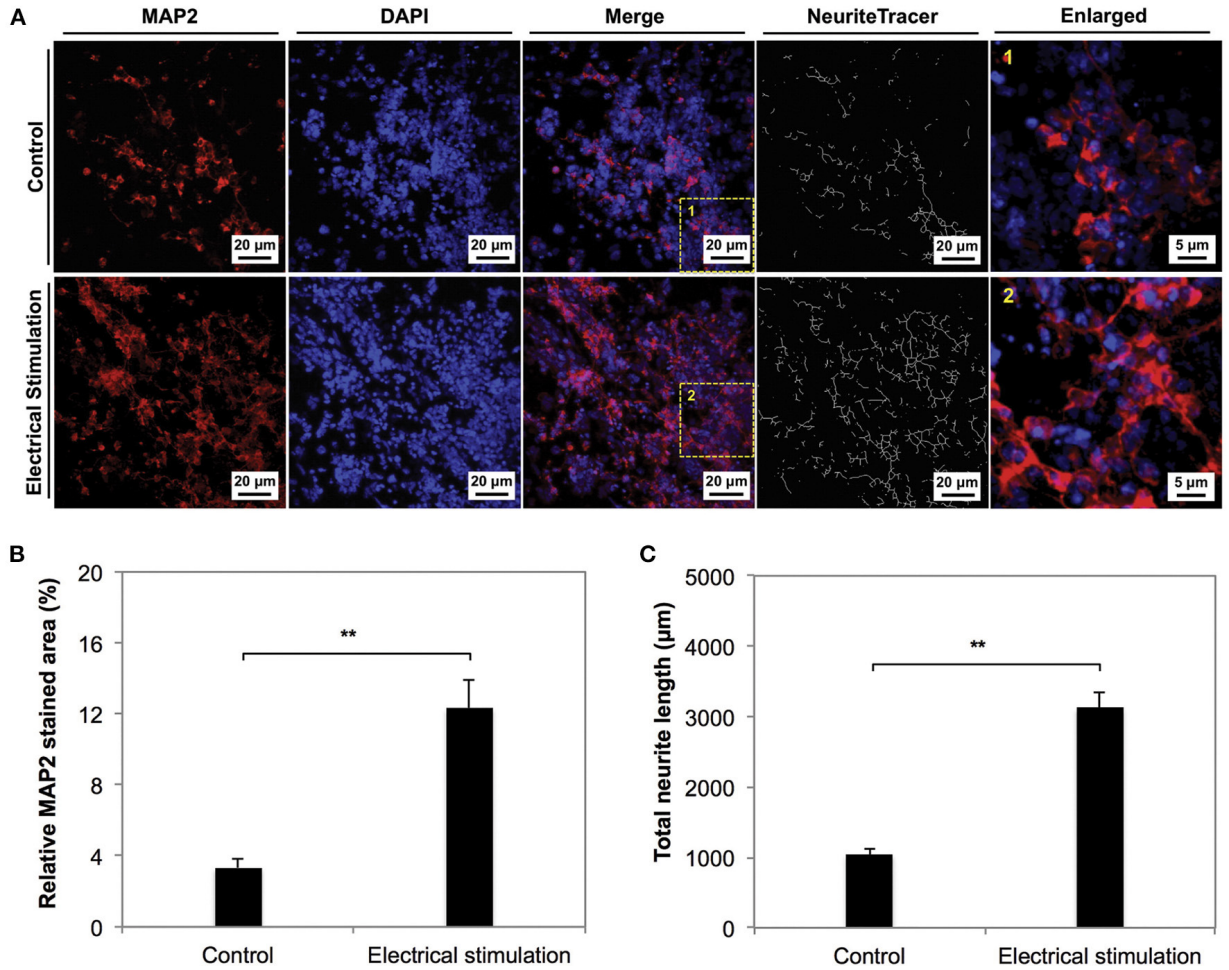
The phenotype of NSCs cultured on electrospun carbon nanofibers under electrical stimulation was compared to unstimulated controls for 7 days in culture. **(A,B)** The neuronal marker MAP2 was significantly expressed in the stimulated condition compared to the control. **(C)** Neurite length anaylsis indicated longer neurite outgrowth in stimulated conditions. Data was reported as mean ± standard deviation, *n* = 3, **P < 0.01. Reprinted from Zhu et al. (174) with permission from Elsevier.

In addition to the impact on differentiation, ES appears to be cue for enhancing proliferation. Zhu et al. (174) reported a 35% increase in proliferation of NSCs cultured on electrospun carbon nanofibrous scaffolds, proposed to occur *via* proliferating cell nuclear antigen and extracellular signal-regulated kinases 1 and 2 mechanisms (175). Furthermore, the group concluded that ES increased the neuronal differentiation of hNSCs, with complex morphologies reported (Figure 6). More recently, Song et al. (176) demonstrated changes in protein expression including heparin binding EGF-like growth factor (hb-EGF), GDNF, BDNF and neurotrophin 3 (NTF3) and enolase 2 (ENO2) on human NPCs as a result of the change of dimensionality from 2D to 3D PPY scaffolds. The group linked these gene expression changes to enhanced cell survival. These studies have provided a basis to incorporate conductivity and ES into tissue engineering applications to enhance neural regeneration from the functional observations of improved cellular proliferation and neuronal differentiation.

CNTs, graphene and fullerenes, are nanomaterials with interesting optoelectronic and mechanical properties which have been incorporated in electroactive biomaterials. Reports in the literature indicate the potential of electroactive biomaterials may enhance survival, facilitate cell-cell adhesion, proliferation, differentiation and immune homeostasis, that are pertinent to neural regeneration (168, 177) (Table 3). The novelty of combining carbon-based electroactive material surface topography with a polymer and ES has been demonstrated with promising results for enhancing neural regeneration *in vitro*. Key findings by Shin et al. (178) indicate significant neuronal differentiation of human fetal neural stem cells (hfNSCs) and human induced pluripotent stem cell-derived neural progenitor cells (hiPSC-NPCs). Furthermore, improved electrophysiological functionality within catechol-functionalized hyaluronic acid (HA–CA) hydrogels containing CNTs and/or PPy was reported in this study. A recent *in vitro* study reported the feasibility of electrically stimulating NSCs on graphene oxide (GO)-incorporated poly(lactic-co-glycolic acid) (PLGA) electrospun nanofibers (179). The authors reported the enhanced proliferation, neuronal differentiation and neurite outgrowth of NSCs, similar to findings by Zhu et al. (174) thus indicating the potential of applying graphene oxide composites, albeit in a concentration-dependent manner due to reports of cytotoxicity, as electroactive scaffold implants to promote neurogenesis (180). The authors further demonstrated the feasibility of immobilizing insulin-like growth factor 1 (IGF-1) on GO with significant improvement in NSC survival outcomes, indicating further potential for incorporating nanoparticle drug delivery within these composite electroactive scaffolds (162). Combining electroactivity, ES and nanomaterial science to neural tissue engineering adds a level of complexity to scaffold biomaterial design for neural regeneration applications. The combined effects of these concepts show promise for an implantable electroactive scaffold as an effective combinatorial approach in SCI cell therapy research.

There are considerable challenges to overcome before clinical application of an electroactive cell implant can become a reality, such as control over mechanical properties to affect cell-cell interactions, responses and integration with the host tissue. In order to optimize their application in the CNS, the porosity and microarchitecture of hydrogels requires significant control. This has partly been achieved through traditional polymer processing techniques as hydrogel scaffolds of uniform porosity have successfully been developed, and even progressed to clinical trials for neural tissue repair as reported by Theodore et al. (181). This group demonstrated the safety and feasibility of the first human implantation of a porous bioresorbable polymer scaffold into the acutely contused spinal cord. Clinical efficacy has not yet been achieved, and despite promising results in preclinical models, hydrogel scaffolds require further development as the mechanical properties have proven difficult to control. Researchers like Prager et al. (182) have brought innovation to this area by reporting a protocol to determine a “target stiffness” for hydrogels, matched to the stiffness of the CNS, indicating development of the strategy for neural tissue repair. The application of electroactive hydrogel scaffolds and ES for SCI cell therapy, too, requires further development from the preclinical evidence gathered so far.

## Future Directions

This review highlights the effects of electroactivity and ES on NSCs in 3D matrices, largely composed of *in vitro* preclinical reports. There is clear potential for these scaffolds to be used to modulate NSC behavior post-transplantation and enhance their regenerative capability. However, there are gaps in our current understanding. Much of the current data is focused on neurogenesis and neuronal maturation. Whilst important, the glial components of the CNS are also essential to its function and pathological response to injury. Therefore, systematic studies into the effect of electroactive scaffolds on glial cell production from NSCs are needed. Further, understanding the effect of electroactive materials and ES on microglia will be key to engineering the immune response to the implant, a major factor in successful implant integration and repair capacity.

The control of mechanical properties of electroactive scaffolds, such as stiffness, appears to be a challenge. The development of novel electroactive hydrogel scaffold composites incorporated with microfabrication techniques and nanomaterials may partly address this limitation (168). Whilst there is a growing body of *in vitro* evidence for the cytocompatibility of implants such as graphene-based composite scaffolds, standardized studies in animals with the objective of assessing their nanotoxicology seem necessary to determine the clinical translation of such developments. Some recent preclinical advances demonstrate the long term performance of electroactive scaffolds for neural tissue regeneration applications *in vivo* (183, 184). Thus the main hurdles to overcome appear to be in the domain of the biocompatibility and safety of electroactive scaffolds *in vivo*, which requires further assessment across different tissues. This would form an essential milestone for progression to clinical translation for a SCI cell therapy. Together with the latter analysis of the insights into electroactive scaffolds, the approvals process and regulatory requirements for medicines and medical devices must be considered to realistically endeavor the clinical translation of electroactive biomaterials for SCI cell therapy. The process is lengthy and often difficult, taking up to 15 years to market a new medicine/medical device. There are many points at which an implantable biomaterial/medical device may be rejected on the grounds of safety, effectiveness and cost over the course of preclinical studies, clinical trial phases, and nation-specific regulatory systems. Therefore, it is essential to carefully probe insights into the long term interaction of implantable electroactive scaffolds with neural tissue at the current preclinical stage.

Degradable electroactive hydrogelsalso have promise for eventual clinical translation. Xu et al. (185) in particular present this rationale in an *in vivo* study testing an injectable electroactive hydrogel composed of biodegradable germanium phosphide (GeP) nanosheets and hyaluronic acid (HA). New technologies could therefore mean translation of implantable electroactive biomaterials for NSC therapy is more realistic. Overall, the combination of electroactive implants and NSC transplantation is a complex therapeutic option. However, given the complexity of SCI injury and the social need for a new therapy we believe this strategy is worth investigating further.

## Author Contributions

AM: methodology and writing—original draft preparation. CA: supervision. JH and CA: project administration and funding acquisition. All authors read, conceptualization, writing—review and editing, and agreed to the published version of the manuscript.

## Funding

AM is supported by a Ph.D. studentship from the Faculty of Natural Sciences at Keele University. JH thank the UK Engineering and Physical Sciences Research Council (EPSRC) for financial support (*via* grants EP/R003823/1, EP/R511560/1, and EP/K03099X/1), the UK Biotechnology and Biological Sciences Research Council (BBSRC) for financial support (*via* grant BB/L0137971/1), and the UK Royal Society for financial support (*via* grant RG160449). CA thanks the UK EPSRC for financial support (*via* an ETERM Fellowship, grant EP/I017801/1) and the Royal Society for financial support (*via* grant RGS/R2/180328).

## Conflict of Interest

The authors declare that the research was conducted in the absence of any commercial or financial relationships that could be construed as a potential conflict of interest.

## Publisher's Note

All claims expressed in this article are solely those of the authors and do not necessarily represent those of their affiliated organizations, or those of the publisher, the editors and the reviewers. Any product that may be evaluated in this article, or claim that may be made by its manufacturer, is not guaranteed or endorsed by the publisher.
